# Differently PEGylated Polymer Nanoparticles for Pancreatic Cancer Delivery: Using a Novel Near-Infrared Emissive and Biodegradable Polymer as the Fluorescence Tracer

**DOI:** 10.3389/fbioe.2021.699610

**Published:** 2021-06-29

**Authors:** Huazhong Cai, Yanxia Chen, Liusheng Xu, Yingping Zou, Xiaoliang Zhou, Guoxin Liang, Dongqing Wang, Zhimin Tao

**Affiliations:** ^1^School of Medicine, Jiangsu University, Zhenjiang, China; ^2^The Affiliated Hospital, Jiangsu University, Zhenjiang, China; ^3^College of Chemistry and Chemical Engineering, Molecular Imaging Research Center, Central South University, Changsha, China; ^4^Department of Neurology, Xiangya Hospital, Central South University, Changsha, China; ^5^Research Institute for Cancer Therapy, The First Affiliated Hospital, China Medical University, Shenyang, China

**Keywords:** PEGylation, polymer, nanoparticles, near-infrared fluorescence imaging, pancreatic cancer

## Abstract

In this study, a chemically synthetic polymer, benzo[1,2-*b*:4,5-*b*′]difuran(BDF)-based donor–acceptor copolymer PBDFDTBO, was individually coated by amphiphilic poly(ethylene oxide)-block-poly(ε-caprolactone) (PEO-PCL) and 1,2-distearoyl-sn-glycero-3-phosphoethanolamine-N-methoxy(polyethylene glycol) (DSPE-PEG or PEG-DSPE), to form stably fluorescent nanoparticles in the near-infrared (NIR) window. The physicochemical properties of the synthesized nanoparticles were characterized and compared, including their size, surface charge, and morphology. In addition, *in vitro* studies were also performed using two pancreatic cancer cell lines, assessing the cell viability of the PBDFDTBO-included PEGylated nanoparticles formulations. Moreover, *in vivo* studies were also conducted, using subcutaneous murine cancer models to investigate the polymeric nanoparticles’ circulation time, tumor accumulation, and preferred organ biodistribution. The overall results demonstrated that even with the same PEGylated surface, the hydrophobic composition anchored on the encapsulated PBDFDTBO core strongly affected the biodistribution and tumor accumulation of the nanoparticles, to a degree possibly determined by the hydrophobic interactions between the hydrophobic segment of amphiphilic polymers (DSPE or PCL moiety) and the enwrapped PBDFDTBO. Both PEGylated nanoparticles were compared to obtain an optimized coating strategy for a desired biological feature in pancreatic cancer delivery.

## Introduction

Pancreatic cancer remains to be one of the most challenging diseases worldwide. Pancreatic ductal adenocarcinoma (PDAC) arises in ductal epithelium of exocrine pancreas, most diagnosed only at a late stage and thereby not being resectable upon diagnosis (Kleeff et al., 2016). In the United States in 2020, 57,600 new cases are expected to develop and 47,050 would die from this aggressive disease, making it the fourth leading death cause, with a 5-year survival rate after diagnosis less than 5% (Bengtsson et al., 2020; Zavala et al., 2021). Despite the technology advances, the poor prognosis of PDAC has remained unimproved in the past two decades. This seemingly untamable nature of PDAC mainly results from the unusual scarcity of specific biomarkers for diagnosis and the usual resistance to chemotherapeutic reagents during the treatment.

In this context, gemcitabine is a chemically nucleoside analog that is used as a first-line treatment in patients with pancreatic cancer, who previously performed a tumor resection. This strategy approach for the treatment has a reported survival of about 6 months on average (Burris et al., 1997). This survival advantage of postoperative gemcitabine treatment was only observed in patients with lymph node metastases (Skau Rasmussen et al., 2019). Gemcitabine in combination with either erlotinib (a tyrosine kinase inhibitor) or protein-bound paclitaxel (a mitotic inhibitor) did not add a significant benefit to the patient, although additional 1- or 2-month survival could be prolonged with a costly treatment (Moore et al., 2007; Von Hoff et al., 2013). Lately, compared to single gemcitabine therapy, a chemotherapeutic cocktail (FOL-F-IRIN-OX) containing folinic acid (leucovorin), 5-fluorouracil, irinotecan, and oxaliplatin substantially prolonged the patient survival with the diagnosis of metastatic pancreatic cancer (11.1 versus 6.8 months) (Conroy et al., 2011). However, this new chemotherapy regimen became only treatable for patients with good physical status and high tolerance to the drugs’ toxicity.

Ineffective diagnosis and treatment could be highly associated with extraordinary heterogeneity in pancreatic tumor microenvironment. Pancreatic tumors are enclosed by a dense stroma, including a diversity of cellular and acellular substances, with the pancreatic cancer cells being hardly accessible *via* conventional pharmaceutical delivery (Liang et al., 2017; Hosein et al., 2020). In addition, unlike many other angiogenic cancers, which developed irregular blood vessels *de novo*, PDAC is histologically characterized as poorly vascularized (Ryan et al., 2014). Therefore, uncommon richness of stroma and deficiency of vasculature, plus common presence of hypoxia in PDAC, are responsible to further reduce the intratumoral blood flow, elevate the interstitial pressure and decrease delivery of therapeutic drugs to tumor sites through the bloodstream (Ryan et al., 2014). Thus, an effective and specific delivery of theranostic agents to overcome the stromal barrier and target the pancreatic cancers became imperative to precisely diagnose and specifically treat PDAC (Han et al., 2018; Hu et al., 2021).

Nanomedicine has shown a unique and unmatched privilege of their enhanced permeability and retention in pancreatic cancers with hypovascular and poorly permeable features (Cabral et al., 2011; Meng et al., 2015). In fact, facile conjugation of various targeting moieties, according to different cancer origins, has enabled the specified and concentrated nano-drug delivery (Zhang et al., 2021). Among them, polymeric nanoparticles have been widely adopted in biomedical and clinical research because of their organic nature and optimal properties, such as bioavailability, biocompatibility, and biodegradability (Tao et al., 2013; Hong et al., 2014). In particular, the surface modification of nanoparticles by using polyethylene glycol (PEG) has been adopted as the most common strategy to prolong the nanoparticles’ blood circulation and eliminate immunogenicity, therefore increasing their accumulation in the targeted organs or tumors (Suk et al., 2016).

Here, two different PEGylated amphiphilic polymers were selected to modify one novel near-infrared (NIR) emissive and biodegradable polymer for fluorescent imaging of pancreatic cancers in two subcutaneous murine models. A donor unit benzo[1,2-b:4,5-b′]difuran(BDF)-based donor–acceptor (D–A) copolymer PBDFDTBO was synthesized (Liu et al., 2012; Gao et al., 2014) and applied in this study for the first time as a fluorescent probe with bright and stable emission in NIR-I window. Choosing this polymeric dye rather than the small-molecule dye is mainly due to the following reasons: (i) the polymeric dye with a conjugated system owns a larger Stokes shift, which results in much better signal-to-noise ratio (Dutta et al., 2009; Ren et al., 2018); (ii) the polymeric dye has much enhanced photostability in a variety of chemical environments with improved resistance to photobleaching (Mao et al., 2015); (iii) the polymer with high molecular weight usually enables a longer circulation time *in vivo* than the small-molecule substance (Ghezzi et al., 2021). Moreover, due to the hydrophobic nature of PBDFDTBO polymer, we employed the commonly adopted PEGylation strategy to modify it for a good availability in the biological system. Thus, 1,2-distearoyl-sn-glycero-3-phosphoethanolamine-N-methoxy(polyethylene glycol) (PEG-DSPE; Bedu-Addo and Huang, 1995) or poly(ethylene oxide)-block-poly(ε-caprolactone) (PEO-PCL; Grossen et al., 2017) were chosen to differently PEGylate PBDFDTBO to compare their biological features for pancreatic cancer delivery. Intriguingly, it also remains unexplored whether the hydrophobic segment in PEG-based amphiphilic polymers has a modulatory effect on their pharmacokinetics, biodistributions, and tumor accumulations. The results would help reveal an optimized PEGylation strategy for nanoparticulate delivery into pancreatic tumors.

## Materials and Methods

### Materials, Cells, and Animals

PEO(5000)-b-PCL(5000) and PEG(5000)-DSPE were purchased from Xi’an Ruixi Biological Technology Co., Ltd. (Xi’an, China) and Avanti Polar Lipids Inc. (Alabaster, AL, United States), respectively. PBDFDTBO (P for short in the following figures) was synthesized and characterized as previously reported (Liu et al., 2012). Tetrahydrofuran (THF) and phosphate-buffered saline (PBS) were purchased from Vicente Biotechnology Co., Ltd. (Nanjing, China). Ultrafiltration centrifuge tubes (molecular weight cutoff or MWCO = 100 kDa) were purchased from Millipore (MA, United States). Cell Counting Kit-8 (CCK-8) assay was obtained from Dojindo (Kumamoto, Japan). Mouse pancreatic cancer cell line Panc02 was obtained from Chinese Academy of Sciences (Shanghai, China) and cultured in high glucose Dulbecco’s Modified Eagle’s Medium (H-DMEM, BioInd) with a 10% fetal bovine serum (FBS, Excell). Human pancreatic cancer cell line PATU-8988T was obtained from Shanghai YuBo Biotech Co., Ltd. (Shanghai, China), being cultured in H-DMEM with 10% FBS. Nude mice (6-week-old female) were purchased from Changzhou Cavins Laboratory Animal Co., Ltd. (Changzhou, China). All the animal experiments were carried out following the guidelines of the Experimental Animal Administrative Committee of Jiangsu University.

### Syntheses of PEO-PCL-P and PEG-DSPE-P Nanoparticles

In typical synthesis, PEO-PCL or PEG-DSPE were dissolved in the THF at a concentration of 20 mg/ml, and 200 μl of the solution (i.e., 4 mg PEG-based amphiphilic polymer) was then pipetted into a glass bottle, mixed thoroughly with 10 μl of 4 mg/ml PBDFDTBO solution in the THF, before the mixture was added dropwise into 5 ml dH_2_O during ultrasonication with the energy of 800–900 Joule applied continuously for 1 min to form the nanoparticles. Subsequently, the mixture was transferred to the ultrafiltration tube (MWCO = 100 kDa) and centrifuged at 1500 *g* for 30 min to remove the organic solvent. The residues were washed three times by PBS to ensure the complete removal of the organic solvent. The obtained nanoparticles were dissolved in 200 μl of PBS and filtered using a 0.22-μm polyethersulfone filter (Sigma-Aldrich, United States) for sterilization purposes before *in vitro* or *in vivo* experiments.

### Characterizations of Polymeric Nanoparticles

One hundred microliters of the synthesized PEO-PCL-P or PEG-DSPE-P nanoparticles was added to a 96-well plate (CellStar, United States). First, the absorption wavelength of the two particles was measured by the microplate reader (CellStar, United States) and then the absorption peaks were determined. The corresponding wavelength under peak absorbance was selected as the excitation wavelengths to further acquire the emission spectra. The size and ζ-potential of PEO-PCL-P or PEG-DSPE-P nanoparticles were measured by Nanoparticle Tracking Analysis (NTA ZetaView^®^PMX120) or Zetasizer Nano Analyzer (Malvern ZS90). A cryogenic transmitting electron microscopy (cryo-TEM) observation of polymeric nanoparticles in solutions was carried out in a controlled-environment vitrification system. The climate chamber temperature was 25–28°C, and the relative humidity was kept close to saturation to prevent the sample evaporation during the preparation. The samples at room temperature were placed on a carbon-coated holey film supported by a copper grid and gently blotted with filter paper to obtain a thin liquid film (20–200 nm) on the grid. The grid was quenched rapidly in liquid ethane at −180°C and then transferred to liquid nitrogen (−196°C) for storage. Then, the vitrified specimen stored in liquid nitrogen was transferred to a Tecnai G2 F20 cryo-microscope, using a Gatan 626 cryoholder and its workstation. The acceleration voltage was 200 kV, and the working temperature was kept below −170°C. The images were digitally recorded with a charge-coupled device camera (Gatan) under low-dose conditions.

### Cytotoxicity Tests

The pancreatic cancer cell lines, 4000 Panc02 cells/well and 6000 8988T cells/well, were placed into 96-well plates and six duplicate wells were set for each condition overnight, before the supernatant was then carefully discarded and the freshly cell culture solutions with pre-added PEO-PCL-P and PEG-DSPE-P nanoparticles at different concentrations were added. The cell plates were gently shaken and moved to the incubator for 24-h treatment. For the measurements, the supernatants were removed by leaving adherent cells undisturbed, and the fresh culture solutions containing 10% CCK8 reagent were next supplied for 2 h incubation at 37°C. The absorbance of each well was measured in a microplate reader.

### *In vivo* Animal Experiments

Six-week-old female nude mice were subcutaneously injected with 100 μl of 5.0 × 10^7^/ml Panc02 cells or 5.0 × 10^7^/ml 8988T cells on both left and right flanks during anesthesia. Mice were closely monitored after the tumor cells were implanted, and animal experiments were initiated when the tumor size reached ∼0.5–0.8 cm in diameter. PEO-PCL-P or PEG-DSPE-P nanoparticles were injected into the tail vein and mouse blood was sampled at the time points as indicated. At the same time, the fluorescence intensity of the tumor on the left and right sides of nude mice was also measured when co-localized through bright field observations of subcutaneous tumor regions. At the end of animal experiments, mice were sacrificed, and the major organs were extracted and measured for their *ex vivo* fluorescence distributions. Animal experiments were performed using IVIS^®^ Spectrum *In Vivo* Imaging System (PerkinElmer, United States).

### Statistical Analysis

All data were shown as the mean ± standard deviation (SD). All statistical analysis was performed with GraphPad Prism 5.0 software (GraphPad Software Inc., United States). The statistical differences between groups were analyzed using Student’s *t*-test. A *p*-value of <0.05 was considered to indicate a statistically significant difference.

## Results

### Synthesis of NIR-I Emissive Biodegradable Nanoparticles

**FIGURE 1:**
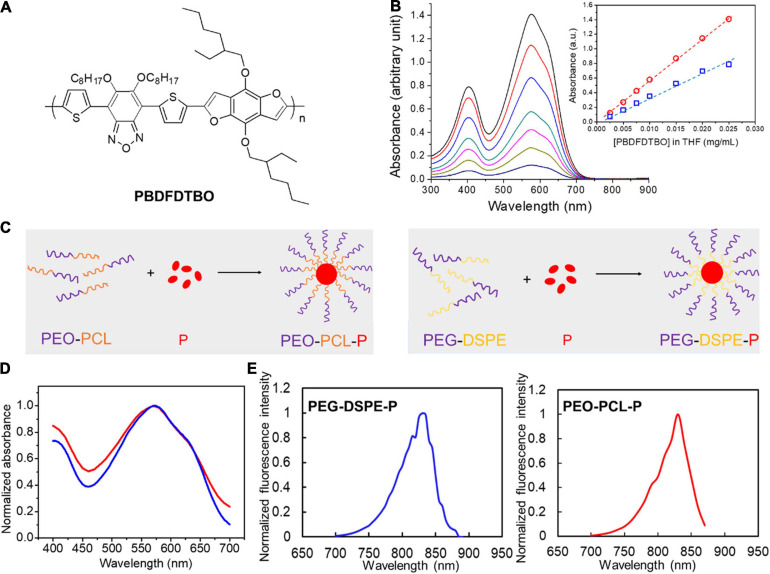
Chemical structure of PBDFDTBO and photoluminescent properties of PBDFDTBO and PBDFDTBO-included PEGylated nanoparticles. **(A)** Chemical structure of PBDFDTBO. **(B)** UV-VIS spectra of PBDFDTBO in THF solutions at different concentrations, with insert graph showing absorbance peak at 401 nm (blue) and at 574 nm (red) plotted versus the according concentration of PBDFDTBO. **(C)** Schematic illustration of PEO-PCL-P (left) and PEG-DSPE-P (right) nanoparticle synthesis. **(D)** Absorption spectra of PEO-PCL-P (red) and PEG-DSPE-P (blue) nanoparticles in aqueous solutions, where absorbance intensity was normalized to the peak absorbance value in each curve. **(E)** Fluorescence spectra of PEO-PCL-P (red) and PEG-DSPE-P (blue) nanoparticles in aqueous solutions, where fluorescence intensity was normalized to the peak value in each spectrum.

The chemical structure of PBDFDTBO (P) is represented in Figure 1A. At first, different concentrations of PBDFDTBO were prepared in THF and measured for their absorbance in a range of wavelength from 300 to 900 nm, with findings that this polymeric substance has two absorbance peaks, one at 401 nm and the other at 574 nm (Figure 1B). Peak values were plotted versus the corresponding PBDFDTBO concentrations, exhibiting an excellent linearity (*R*^2^ > 0.99). Due to the strong hydrophobic nature of PBDFDTBO, we adopted two PEG-based amphiphilic polymers to encapsulate this D–A copolymer inside the hydrophobic core, making it more water-soluble. The synthetic scheme was illustrated in Figure 1C. PEG-DSPE or PEO-PCL was dissolved in THF, where PBDFDTBO solution was added to mix well before these compounds were dispersed into dH_2_O during sonication. Organic solvents were later removed, and the synthesized particles were resuspended in aqueous solutions (i.e., PBS) for the further characterizations. Absorption spectra of both synthesized PEO-PCL-P and PEG-DSPE-P nanoparticles showed the highest absorbance peak at 570 nm (Figure 1D). Both nanoparticle solutions demonstrated fluorescence emission spectra Figure 1E, where substantial emission emerged since 750 nm with peak values at 830 nm, using 570 nm as an excitation wavelength.

**FIGURE 2:**
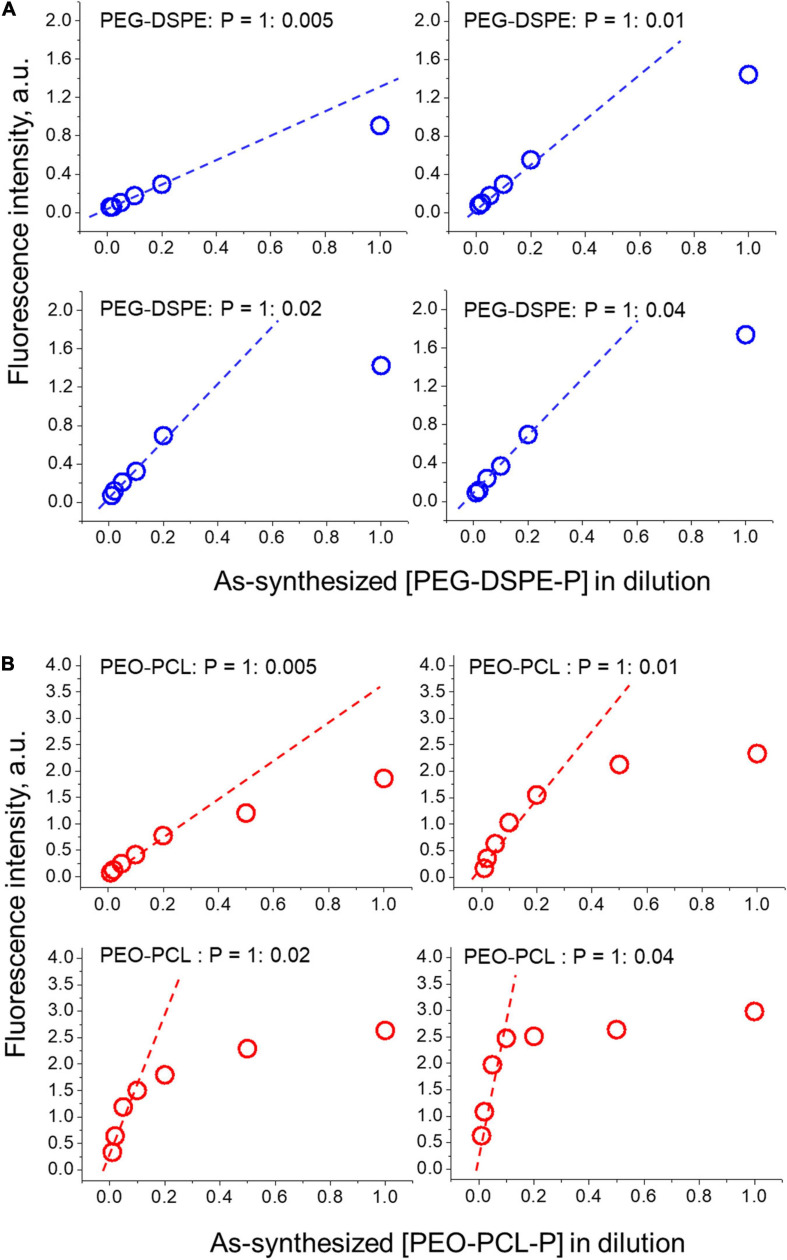
Optimization of PBDFDTBO encapsulation by differently PEGylated nanoparticles **(A)** PEG-DSPE-P and **(B)** PEO-PCL-P. Initial mass ratio between polymer (PEO-PCL or PEG-DSPE) and PBDFDTBO = 1: 0.005, 0.01, 0.02, and 0.04 was applied and the synthesized dye-contained nanoparticles were diluted in PBS with a dilution factor = 2, 5, 10, 20, 50, and 100, respectively, when fluorescence intensity of each solution was measured and plotted versus nanoparticle concentrations.

To optimize the encapsulation of PBDFDTBO in the amphiphilic polymer, different initial concentrations of this D–A copolymer were employed with a fixed amount of PEO-PCL or PEG-DSPE, to prepare water-soluble nanoparticles and test their fluorescent properties in different dilutions. The results were summarized and shown in Figure 2. With an initial mass ratio between amphiphilic polymer (PEO-PCL or PEG-DSPE) and PBDFDTBO = 1: 0.005, 0.01, 0.02, and 0.04, respectively, nanoparticles were synthesized as shown in section “Materials and Methods” and diluted in PBS with a dilution factor = 2, 5, 10, 20, 50, and 100, respectively. Fluorescence intensity of each formulation was measured and plotted versus the concentration of nanoparticles (in dilution as shown in Figure 2). As a result, the mass ratio of PEO-PCL or PEG-DSPE: PBDFDTBO = 1:0.01 was chosen for the nanoparticle synthesis, due to the finding that after 10 times dilution of as-synthesized nanoparticles in aqueous solutions, fluorescence intensity became linearly altered when nanoparticle concentration changed (a standard injection of 200 μl *via* mouse tail vein would be diluted about 10 times by presuming the whole mouse blood volume of 2 ml). In this range, detecting the fluorescence intensity of nanoparticles represents a valid measure in evaluating the nanoparticle of an unknown concentration.

### Characterization of Differently PEGylated Nanoparticles

To calculate the composition of as-made nanoparticles, nanoparticles after typical synthesis were lyophilized and re-dissolved in THF, and the PBDFDTBO contained in PEO-PCL-P or PEG-DSPE-P was measured at 5.4 ± 0.3 and 7.4 ± 1.5 μg/mg nanoparticles. At the same time, the as-synthesized PEO-PCL-P or PEG-DSPE-P after typical synthesis and purification was measured at 3.6 ± 0.3 and 2.7 ± 0.2 mg, respectively; that is, 18.0 ± 1.5 mg/ml PEO-PCL-P and 13.5 ± 1.0 mg/ml PEG-DSPE-P in 200-μl aqueous solutions.

The PEO-PCL-P or PEG-DSPE-P nanoparticles were then resuspended in dH_2_O, PBS, or serum at the same concentration as above, and tested for their photostability in different chemical environments. By normalizing each fluorescence intensity under all the tested condition (*n* = 5) to the highest one acquired, the results were shown in mean ± SD in Supplementary Figure 1A. For PEG-DSPE-P nanoparticles, the normalized fluorescence intensity became 0.97 ± 0.02, 0.96 ± 0.02, and 0.93 ± 0.01 in dH_2_O, PBS, and serum solutions, respectively. For PEO-PCL-P nanoparticles, the normalized intensity read was 0.98 ± 0.02, 0.98 ± 0.01, and 0.95 ± 0.02 in dH_2_O, PBS and serum, respectively. Therefore, the fluorescence intensity in serum dropped significantly for both nanoparticles compared with the corresponding results in water or PBS conditions (*p* < 0.05). Then, the synthesized nanoparticles were incubated in serum at 37°C and protected from light for 6 days, and their fluorescence intensity was measured at days 0, 2, 4, and 6, and then normalized to the highest one acquired. Results are shown in Supplementary Figure 1B, as the normalized fluorescence intensity of each nanoparticle formulation was plotted over time. For both nanoparticles incubated in serum solutions, the fluorescence intensity became gradually lower over time and remained 70% of the original value after 6 days suspension. Furthermore, the synthesized nanoparticles in dH_2_O, PBS, or serum were subjected to continuous photobleaching, as formulations were exposed to the excitation of 570 nm and emission intensity at 830 nm was recorded every 30 s. The fluorescence intensity was acquired by being normalized to the highest one under each condition and plotted versus time, as shown in Supplementary Figure 1C. It was observed that the photoluminescence was stable over continuous exposure to excitation light, demonstrating a good resistance to photobleaching.

**FIGURE 3:**
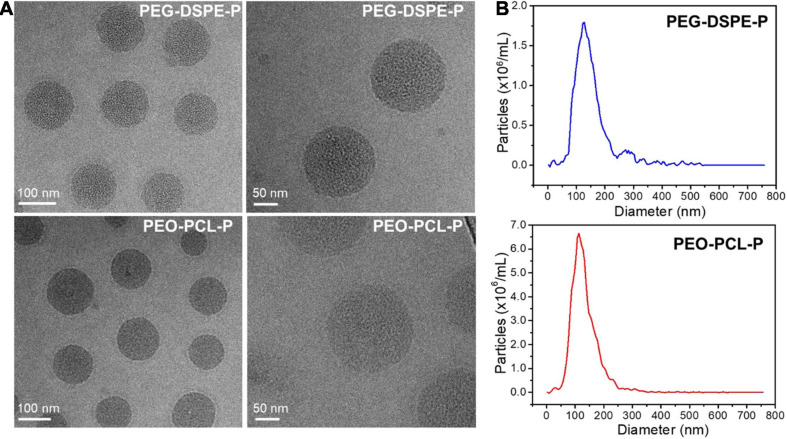
Physicochemical properties of PEO-PCL-P and PEG-DSPE-P nanoparticles. **(A)** Cryo-TEM images of both nanoparticles showing sizes of ∼120 nm with a spherical morphology. **(B)** Size distribution of PEG-DSPE-P (blue) and PEO-PCL-P (red) nanoparticles.

Further characterizations were investigated using TEM and NTA measurements, which corroborated the size, morphology, and surface charge of both nanoparticles (Figure 3). The nanoparticle size of PEO-PCL-P was measured with a peak value of 121 nm, with a surface potential of −24 mV. Similarly, the particle size of PEG-DSPE-P was measured with a peak value of 118 nm with a surface potential of −26 mV. Their hydrodynamic diameters were visualized and verified by cryo-TEM images with a spheric morphology. Particle stability was next tested by suspending PEO-PCL-P or PEG-DSPE-P in dH_2_O and measuring their size, polydispersity index (PDI), and zeta potential over time (0, 2, and 4 days after syntheses, measured by Zetasizer Nano Instrument). Results were shown in Supplementary Figure 1D. Upon synthesis, PEO-PCL-P had a particle size of 126.3 ± 0.8 nm with a PDI of 0.231 ± 0.022 and a zeta potential of −25.3 ± 0.6 mV, whereas PEG-DSPE-P had a particle size of 123.0 ± 5.1 nm with a PDI of 0.224 ± 0.046 and a zeta potential of −29.5 ± 1.6 mV. Compared to PEO-PCL-P, PEG-DSPE-P owns indistinguishable particle size and PDI, but a slightly more negative surface charge. Moreover, during a period of 4 days, both nanoparticles showed great stability in aqueous solutions.

### *In vitro* Toxicity of PEGylated Nanoparticles

The synthesized nanoparticles were studied for their potential cytotoxicity to ensure the biosafety when applying these PEGylated nanoparticles in biological systems. Different concentrations of PEG-DSPE-P or PEO-PCL-P nanoparticles were added into the cell cultures (i.e., murine PDAC cell line PANC02 and human PDAC cell line 8988T) and tested for their toxicity at 24-h incubation. The results are shown in Supplementary Figure 2. For PANC02 cells, dosages of 0–2000 μg/ml nanoparticles were examined for cytotoxicity, in which PEO-PCL-P showed no noticeable toxicity, and PEG-DSPE-P remained non-toxic until 892 μg/ml (cell viability then dropped to 85.4 ± 7.0%). In comparison, the toxicity for 8988T cells showed a much-elevated sensitivity, as dosage of nanoparticles over 442 μg/ml started to lower the cell viability to 92.2 ± 5.1% in the presence of PEG-DSPE-P and to 80.9 ± 5.8% in the presence of PEO-PCL-P nanoparticles (*p* < 0.05 when compared to untreated cells), respectively. Therefore, both PEGylated nanoparticles presented a minimal toxicity to pancreatic cancer cells studied, while for the same nanoparticle, PEO-PCL-P or PEG-DSPE-P, it showed different toxicity profiles to cells with different origins.

### *In vivo* Pharmacokinetics, Biodistribution, and Tumor Accumulation

Mouse (PANC02) or human (8988T) PDAC cells were transplanted into the left and right flanks of nude mice, allowing for a subcutaneous tumor growth of a 2-week period. A tumor with a diameter of approximately 0.5 or 0.8 cm was formed for 8988T or PANC02 cells, respectively. Before *in vivo* administration of nanoparticles, the mouse blood was obtained, and different concentrations of PEO-PCL-P or PEG-DSPE-P were added into the mouse blood for *ex vivo* measurement of fluorescence intensity. Results are shown in Supplementary Figure 3, and it was observed that nanoparticle concentrations less than 10 times dilution of the highest concentration tested (i.e., 18 mg/ml for PEO-PCL-P and 13.5 mg/ml for PEG-DSPE-P) exhibited a good linear function versus fluorescence intensity. Presuming a whole mouse blood volume of 2 ml, 200 μl of 18 mg/ml for PEO-PCL-P and 13.5 mg/ml for PEG-DSPE-P can be the intended injection dosage for the next *in vivo* study. The nanoparticles were then intravenously administered *via* tail-vein injection in the subcutaneous pancreatic cancer models at a dosage of 180 mg/kg mouse for PEO-PCL-P or 135 mg/kg mouse for PEG-DSPE-P. Mouse blood was withdrawn at different time points for the pharmacokinetics study and the polymeric dye-included nanoparticles were checked by their fluorescence intensities in various organs or tumors for biodistribution and tumor accumulation studies.

**FIGURE 4:**
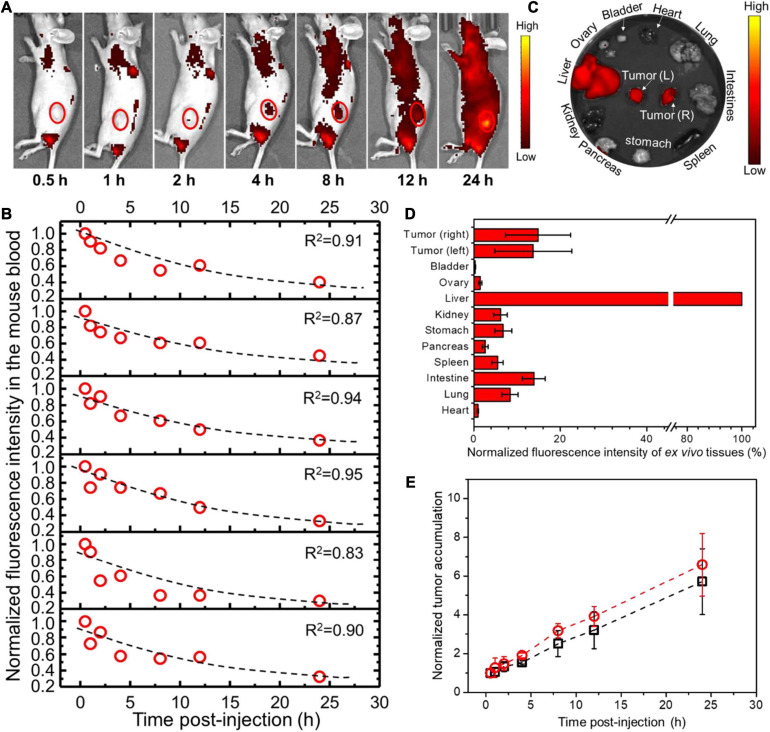
*In vivo* profiles of PEO-PCL-P nanoparticles in PANC02 cell-transplanted subcutaneous tumor models after tail-vein injection. **(A)** Overlapping bright field images and fluorescence images of one representative mouse over time (indicated below the image), depicting the circulation of fluorescent nanoparticles and the accumulation into the observed tumor region (red circle, only showing left flank as example). **(B)** The fluorescence intensity of mouse blood was plotted versus the withdrawal time, fitting into an exponential function (illustrated as a dotted line in each graph panel), so the exponential decay constant was acquired for conversion into the circulation half-time (*t*_1__/__2_). **(C)** At 24 h p.i., the mice were sacrificed, and major organs were imaged. **(D)** The fluorescence intensity of each organ was recorded per tissue area and normalized to that of liver (100%). **(E)** The fluorescence intensities of both left and right tumors were recorded over time, normalized to the first fluorescence reading in each tumor, and plotted against time. The curves were fitted into linear functions, where the slope represents the accumulation rate of nanoparticles in the tumor.

For PANC02 cell-transplanted tumor models, both flanks bearing tumors were monitored by co-localizing the tumor region under the bright field with fluorescent imaging (Figure 4A, only showing left flank as example). The fluorescence intensities of both left and right tumors were recorded over time, being normalized to the first fluorescence reading in each tumor and plotted against time as shown in Figure 4E. Simultaneously, mouse blood was collected at different time points post-injection (p.i.) as indicated in Figure 4B, with the fluorescence intensity of mouse blood plotted versus the withdrawal time, fitting into an exponential function where the exponential decay constant was obtained and converted into the circulation half-time (*t*_1__/__2_). At 24 h p.i., the mice were sacrificed, and the major organs were extracted and analyzed by *ex vivo* fluorescent imaging (Figure 4C). The fluorescence intensity of each organ was recorded per tissue area and normalized to that of liver (the largest organ), as shown in Figure 4D. As a result, *t*_1__/__2_ for PEO-PCL-P nanoparticles in nude mice were calculated to be 14.5 ± 2.4 h. The tumor uptake at the left flank was 13.7 ± 8.9% (normalized to liver uptake) at a rate of 0.20 h^–1^ to reach the highest accumulation, that is, 5.7 ± 1.7 times the original at 24 h. Similarly, the tumor accumulation at the right flank was 14.8 ± 7.5% at a rate of 0.24 h^–1^ to reach the highest accumulation 6.6 ± 1.6 times the original at 24 h.

PEG-DSPE-P nanoparticles were administered into PANC02 cell-transplanted subcutaneous cancer model *via* tail-vein injection, and the experiments were performed as above-mentioned. As a result, *t*_1__/__2_ for PEG-DSPE-P nanoparticles in nude mice was calculated as 12.4 ± 1.4 h (Supplementary Figure 4A). The tumor uptake at the left flank was 19.7 ± 3.9% (normalized to liver uptake) at a rate of 0.06 h^–1^ to reach the highest accumulation 2.3 ± 0.3 times the original at 24 h (Supplementary Figure 4). The tumor accumulation at the right flank was 15.0 ± 5.8% at a rate of 0.06 h^–1^ to reach the highest accumulation 2.4 ± 0.4 times the original at 24 h p.i. (Supplementary Figure 4 and Table 1). PEG-DSPE-P showed a similar circulation time and tumor accumulation, but their tumor accumulation was much slower in comparison to PEO-PCL-P nanoparticles in the same mouse cancer model transplanted with PANC02 cells.

**TABLE 1:** *In vivo* biological properties of PEO-PCL-P and PEG-DSPE-P in two different subcutaneous pancreatic cancer models.

**Nude mice (*n* = 6)**	**PANC02**		**8988T**	
	**PEO-PCL-P**	**PEG-DSPE-P**	**PEO-PCL-P**	**PEG-DSPE-P**
Circulation half-time, t_1__/__2_ (h)	14.5 + 2.4	12.4 ± 1.4	10.6 ± 0.8^Δ^	9.4 ± 1.0^Δ^
Left tumor accumulation (normalized to liver uptake), %	13.7 ± 8.9	19.7 ± 3.9	14.9 ± 2.3	3.8 ± 2.5^Δ^*
The highest accumulation in the left tumor	5.7 ± 1.7	2.3 ± 0.3*	2.0 ± 0.3^Δ^	2.2 ± 0.3
Accumulation rate, h^–1^	0.20 (*R*^2^ > 0.99)	0.06 (*R*^2^ > 0.96)	0.04 (*R*^2^ > 0.89)	0.05 (*R*^2^ > 0.96)
Right tumor accumulation (normalized to liver uptake), %	14.8 ± 7.5	15.0 ± 5.8	13.3 ± 3.3	3.5 ± 0.7^Δ^*
The highest accumulation in the right tumor	6.6 ± 1.6	2.4 ± 0.4*	1.8 ± 0.2^Δ^	1.7 ± 0.2^Δ^
Accumulation rate, h^–1^	0.24 (*R*^2^ > 0.99)	0.06 (*R*^2^ > 0.96)	0.04 (*R*^2^ > 0.94)	0.04 (*R*^2^ > 0.91)

*p < 0.05 compared to the other nanoparticle in the same mouse model; ^Δ^*p* < 0.05 compared to the same nanoparticle in the other mouse model.

The same *in vivo* experiments were next conducted in 8988T cell-transplanted subcutaneous cancer models of nude mice, using PEO-PCL-P (Supplementary Figure 5) or PEG-DSPE-P (Supplementary Figure 6) nanoparticles. The results are summarized in Table 1. *t*_1__/__2_ was calculated as 10.6 ± 0.8 h for PEO-PCL-P and 9.4 ± 1.0 h for PEG-DSPE-P, respectively, each shorter than that of the same nanoparticles in PANC02 cell-transplanted models. Compared to PEO-PCL-P nanoparticles, PEG-DSPE-P nanoparticles achieved a much lower tumor accumulation (normalized by liver uptake) (3.8 ± 2.5 versus14.9 ± 2.3 in the left tumor, and 3.5 ± 0.7 versus 13.3 ± 3.3 in the right tumor). However, the accumulation rate and the highest accumulation between two nanoparticles in this 8988T cancer model were similar to each other.

## Discussion

In this study, a novel benzo[1,2-b:4,5-b′]difuran(BDF)-based D–A copolymer PBDFDTBO was synthesized and applied as a polymeric dye, which owned the emission in the NIR-I range upon excitation at 570 nm. Two differently PEGylated amphiphilic polymers, PEO-PCL and PEG-DSPE, were used to encapsulate the hydrophobic PBDFDTBO in the core to form water-soluble nanoparticles with a stable emission of NIR-I fluorescence upon excitation. The physicochemical properties of the synthesized PEO-PCL-P and PEG-DSPE-P nanoparticles were investigated, showing a close similarity to each other in many physical and chemical parameters, including their size, shape, and surface properties. For human and murine pancreatic cancer cell lines, both PEO-PCL-P and PEG-DSPE-P nanoparticles at the studied dosages exhibited a similar biocompatibility with no apparent toxicity, although the sensitivity to the cell type could be different based on the exact cell origin. In subcutaneous pancreatic cancer models of nude mice, both PEGylated nanoparticles displayed a prolonged circulation time and enhanced tumor accumulation, albeit the accumulation rates and retentions of nanoparticles in tumors were different. In fact, it was observed that PEO-PCL-P nanoparticles possessed a higher accumulation in 8988T cell-transplanted subcutaneous tumor and showed a faster accumulation in PANC02 cell-transplanted subcutaneous tumor than PEG-DSPE-P nanoparticles.

Poly(ethylene oxide)-block-poly(ε-caprolactone) and PEG-DSPE are among the common strategies to modify the hydrophobic substances with hydrophilic PEG on surface, to fabricate the stealth nanoparticles for enhanced blood circulation (Jokerst et al., 2011; Suk et al., 2016). The density and the molecular weight of PEG chains bound to the nanoparticle surface could also contribute to the efficacy of this shielding effect (Li et al., 2021). Here, both PEGylated on their surfaces, PEO-PCL-P and PEG-DSPE-P demonstrated a similar circulation *in vivo* but different profiles of tumor accumulation. External PEG on nanoparticles could minimize their surface energy, increasing their steric distance among nanoparticles, through the hydrogen bonds between repeating ether units and the environment solvent (Jokerst et al., 2011). In this context, the particle aggregation can be minimized. Furthermore, forming “*brush*” conformation rather than “*mushroom*” conformation, stacking of PEG on nanoparticles reduced non-specific cellular uptake by binding to clusterins and growing protein corona in the particle surrounding (Schöttler et al., 2016), which, in turn, suppressed further protein adsorption and nanoparticle-mediated complement activation (Pannuzzo et al., 2020). Notably, for the same nanoparticle (PEO-PCL-P or PEG-DSPE-P), it exhibited a slightly quicker clearance in the bloodstream in 8988T cell-transplanted mouse model than the PANC02-transplanted one. The underlying reason remains unclear, but it is possible that different tumor burdens would influence the essential metabolism of mice, further altering blood flows (Komar et al., 2009). We have observed a much more rapid growth of PANC02 cell-transplanted tumor than that of the 8988T one, suggestive of likely higher metabolic activity and slower blood flow. This deserves further research efforts to understand it more in depth.

Being hydrophobic tails, DSPE as a phospholipid owns a higher affinity to the biological membrane of bilayer than PCL, achieving a faster and easier cellular penetration and transportation (Yu et al., 2013; Zhao et al., 2013). In parallel, hydrolysis of PCL may be accelerated under acidic and alkaline pH (White et al., 2021), making it less stable for PEO-PCL-P nanoparticles when endocytosed and entrapped in the acidic endosomes. However, in the present study, for pancreatic cancers of rich stroma and poor vasculature, PEO-PCL-P nanoparticles exhibited a faster and higher accumulation than PEG-DSPE-P with similar size, shape, and surface charge. This points out a valuable feature that despite of the similar PEGylated surface, hydrophobic interactions between the included PBDFDTBO and the hydrophobic end of amphiphilic PEG-based polymers (DSPE or PCL) might fine-tune the biodistribution of nanoparticles *in vivo*. Although undetermined, the interaction between PCL and PBDFDTBO favored the tumor accumulation in this study. Further experiments are ongoing to uncover the mechanism.

Currently, the nanomedicine research has extended to targeted molecular therapy, involving not only small-molecule chemical drugs but also a variety of biologics, including nucleotide or protein drugs and cellular immunotherapy (El-Zahaby et al., 2019; Li et al., 2020; Gong et al., 2021). These therapies are the important steps in overcoming some of the unique challenges of pancreatic cancers, which prevent conventional treatments from being effective. At the same time, still the most lethal human malignancy, pancreatic cancer harbors a hypoxic and hypovascular extracellular matrix microenvironment with a dense stroma made of proliferating myofibroblasts, collagen, hyaluronic acid, and other component inhabits. Moreover, the factors produced by the stroma might further support tumor survival and growth (Tao et al., 2021). The presence of this stroma is the major barrier against effectively treating pancreatic cancer in patients (Adiseshaiah et al., 2016). For cancers with rich stroma and poor vasculature like PDAC, a rational design on physicochemical characters of polymeric platforms at nanoscale to enhance their drug delivery and accumulation abilities within tumors is a prerequisite and remains in high demand.

## Conclusion

In summary, two types of PEGylated nanoparticles were here compared to conclude an optimized coating strategy for a desired biological feature in pancreatic cancer delivery. With the same PEGylation on the outer surface, hydrophobic segments that anchor onto the encapsulated core may affect the biodistribution and tumor accumulation of the PEGylated nanoparticles, to a degree that could be determined by the hydrophobic interactions between the hydrophobic ends of amphiphilic polymers and the enwrapped substances. This study paves a new path to adjust nanoparticulate systems for enhanced permeability and retention in pancreatic tumors.

## Data Availability Statement

The raw data supporting the conclusions of this article will be made available by the authors, without undue reservation.

## Ethics Statement

The animal study was reviewed and approved by the Jiangsu University Animal Ethics Administration Committee.

## Author Contributions

HC, YZ, GL, and ZT conceived the idea of study. HC, YC, LX, and XZ performed the experiments. HC, YC, LX, YZ, GL, and ZT conducted the data analysis. HC, YC, LX, YZ, XZ, GL, DW, and ZT contributed to the writing of the manuscript and agreed on this submission and publication. All authors contributed to the article and approved the submitted version.

## Conflict of Interest

The authors declare that the research was conducted in the absence of any commercial or financial relationships that could be construed as a potential conflict of interest.
