# Supplementary material for: Differently PEGylated Polymer Nanoparticles for Pancreatic Cancer Delivery: Using a Novel Near-Infrared Emissive and Biodegradable Polymer as the Fluorescence Tracer
Source: Front Bioeng Biotechnol. 2021 Jun 29;9:699610. doi: 10.3389/fbioe.2021.699610 (PMC8276003; doi:10.3389/fbioe.2021.699610)
Supplement: Supplementary file 1 [file Data_Sheet_1.docx]

Supplementary Information

for

**Differently PEGylated Polymer Nanoparticles for Pancreatic Cancer Delivery: Using A** **Novel Near-infrared Emissive and Biodegradable Polymer as the Fluorescence Tracer**

Huazhong Cai^1,2,†^, Yanxia Chen^1,†^, Liusheng Xu^1^, Yingping Zou^3^, Xiaoliang Zhou^4^, Guoxin Liang^5,*^, Dongqing Wang^2^, Zhimin Tao^1,*^

^1^School of Medicine, Jiangsu University, Zhenjiang, Jiangsu 212013, China

^2^The Affiliated Hospital, Jiangsu University, Zhenjiang, Jiangsu 212001, China

^3^College of Chemistry and Chemical Engineering, Molecular Imaging Research Center, Central South University, Changsha, 410083 China

^4^Department of Neurology, Xiangya Hospital, Central South University, Changsha 410008, China

^5^Research Institute for Cancer Therapy, The First Affiliated Hospital, China Medical University, Shenyang, Liaoning 110001, China

^†^Huazhong Cai and Yanxia Chen contributed equally to this work.

^*^Correspondences should be addressed to

Guoxin Liang: [gxliang@cmu.edu.cn](mailto:gxliang@cmu.edu.cn);

Zhimin Tao: [jsutao@ujs.edu.cn](mailto:jsutao@ujs.edu.cn)

**Figure S1.** *In situ* photostability of both nanoparticles in aqueous solutions. (A) PEO-PCL-P or PEG-DSPE-P was resuspended in dH_2_O, PBS or serum and their photoluminescence was recorded. By normalizing each fluorescence intensity under the different conditions (n = 5) to the highest intensity, results were shown in (mean ± standard deviation). (B) The fluorescence intensity of PEO-PCL-P or PEG-DSPE-P nanoparticles in serum was monitored at day 0, 2,4 and 6, when normalized to the highest one acquired. (C) PEO-PCL-P (red) and PEG-DSPE-P (blue) nanoparticles in dH_2_O, PBS or serum were subjected to continuous photobleaching (λ_ex_/λ_em_= 570/830nm), and their fluorescence intensity was recorded every 30 s up to 1 h. The fluorescence intensity was normalized to the highest signal obtained under each condition and plotted versus time. (D) PEO-PCL-P (red) and PEG-DSPE-P (blue) nanoparticles in dH_2_O, being measured for their size, PDI, and zeta potential at time of 0, 2, and 4 days after syntheses.

**
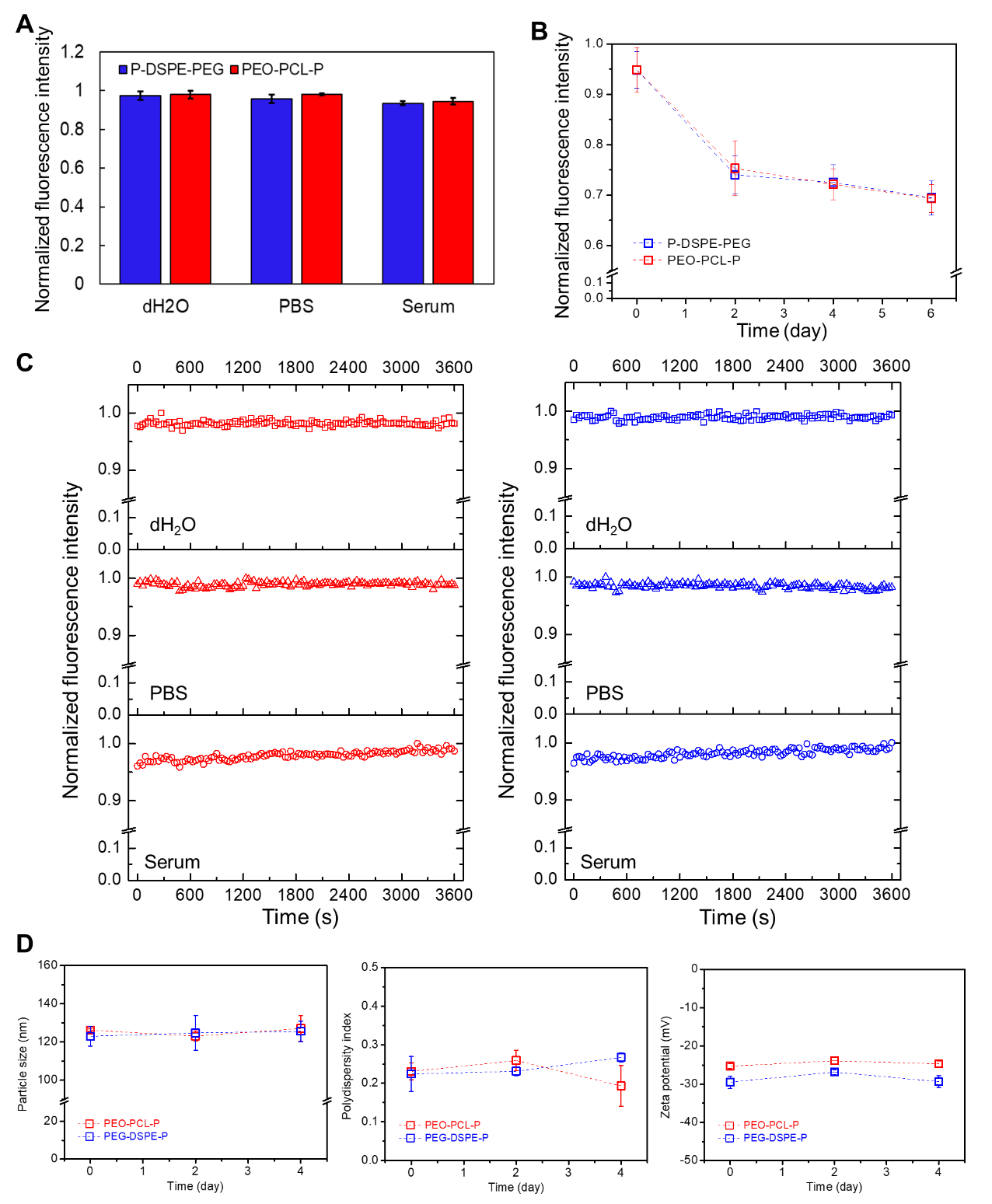
Figure S1.**

**Figure S2.** Cytotoxicity assessment of PEO-PCL-P (red) and PEG-DSPE-P (blue) nanoparticles in two different pancreatic cancer cells lines, namely in PANC02 (A) and 8988T (B).


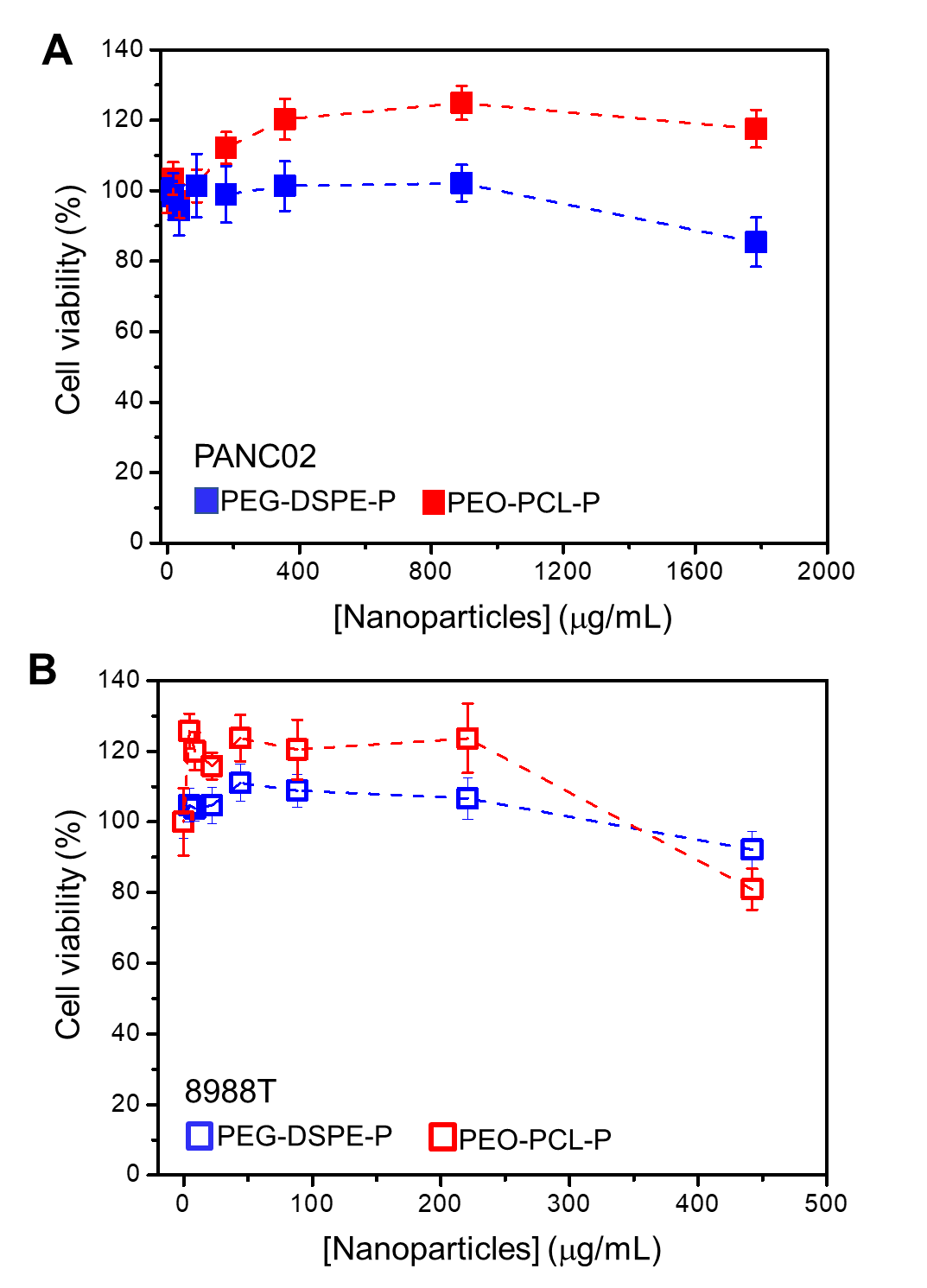


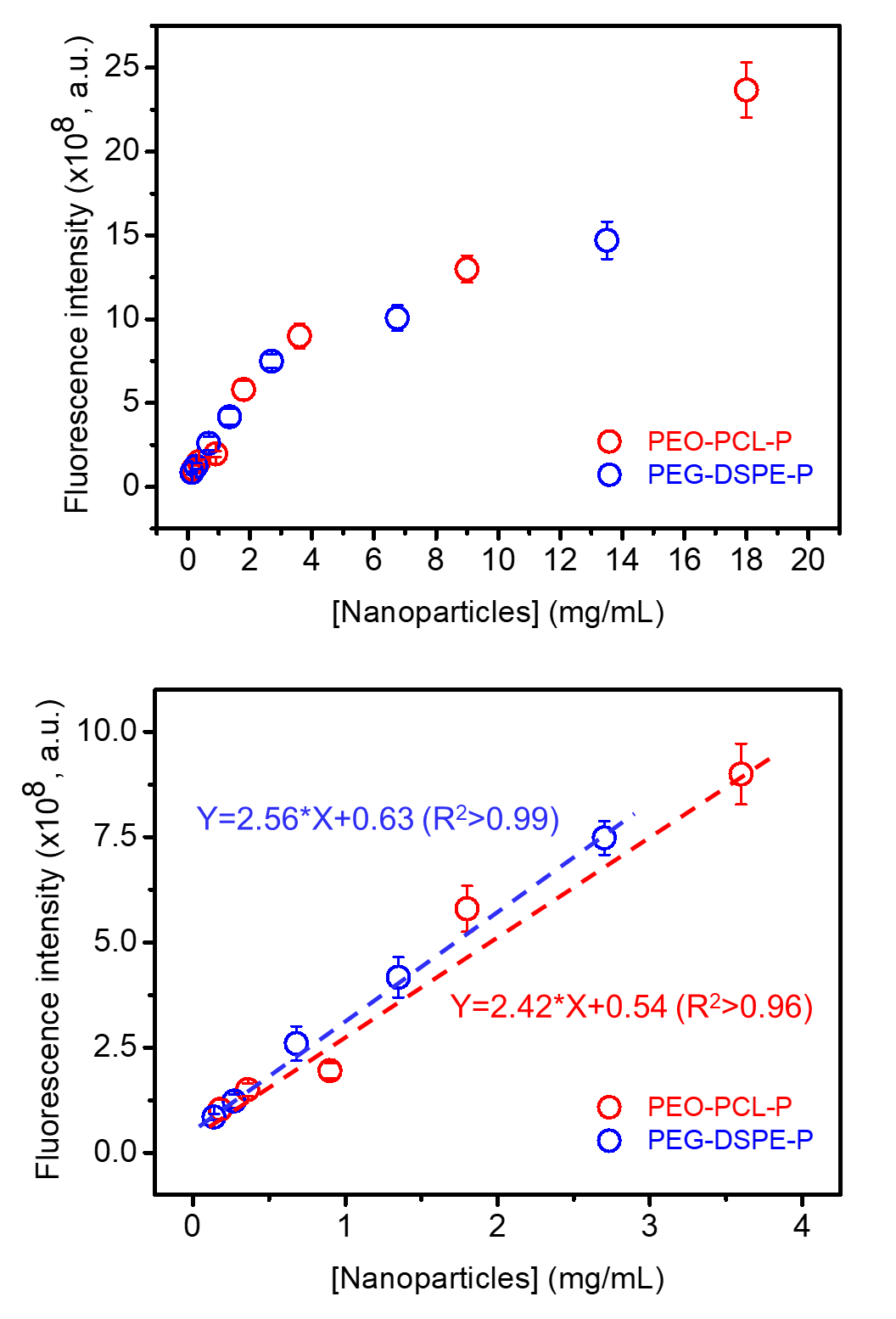
**Figure S3.** Calibration of PEO-PCL-P (red) and PEG-DSPE-P (blue) nanoparticles in the *ex vivo* mouse blood regarding their particle concentration versus fluorescence intensity detected in small animal imaging instrument, to further assess the nanoparticle dosages for *in vivo* administration.


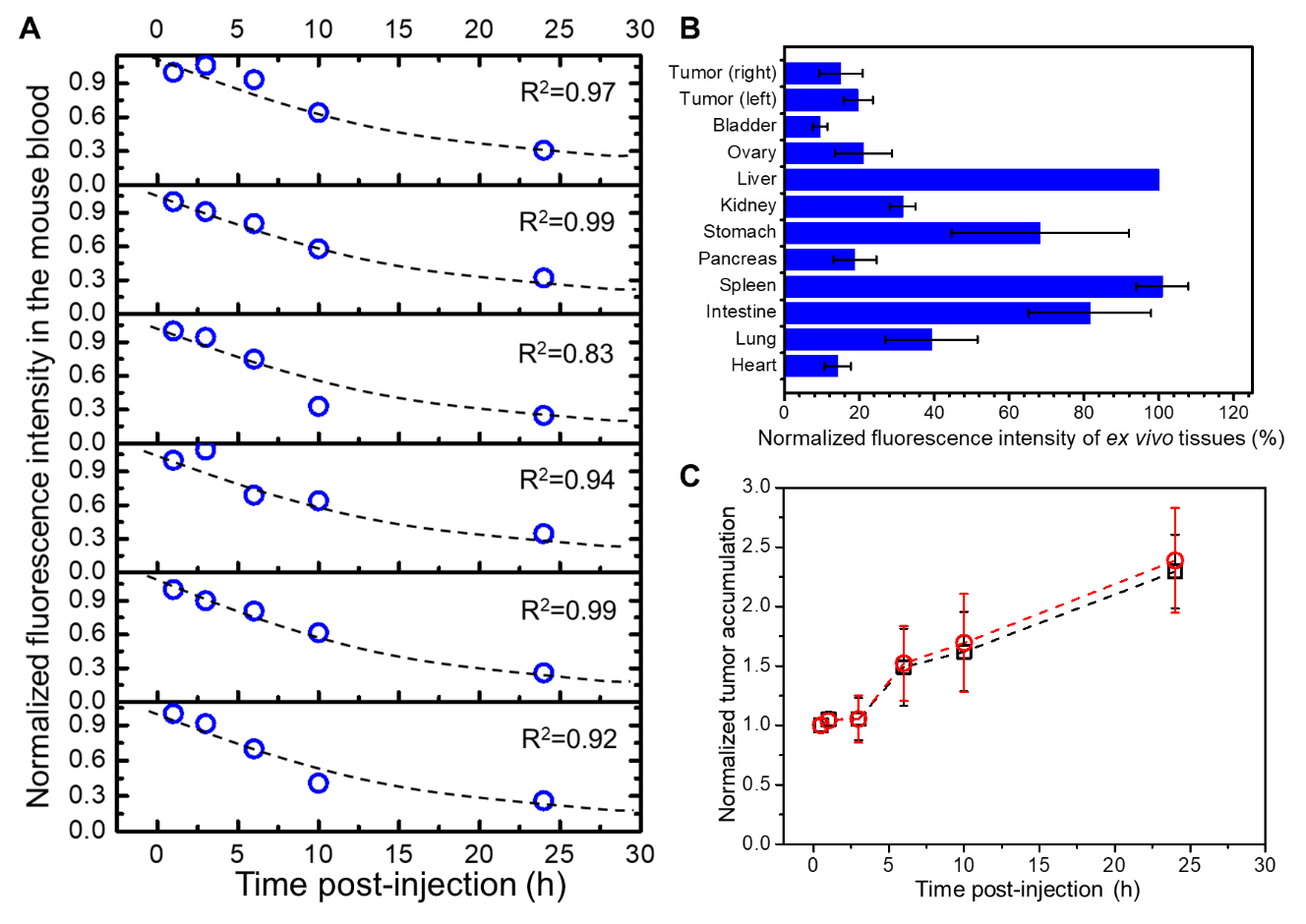
**Figure S4.** *In vivo* results obtained for PEG-DSPE-P nanoparticles in PANC02 cells-transplanted subcutaneous tumor models after tail-vein injection. (A) The fluorescence intensity of mouse blood was plotted versus the withdrawal time, fitting into an exponential function (illustrated as a dotted line in each graph panel). The exponential decay constant was acquired for conversion into the circulation half-time (t_1/2_). (B) At 24 h p.i., the mice were sacrificed, and the fluorescence intensity of each organ was recorded per tissue area and normalized to that of liver (set to 100%). (C) The fluorescence intensities of both left (black empty squares) and right (red empty squares) tumors were recorded over time, normalized to the first fluorescence reading in each tumor, and plotted against time.


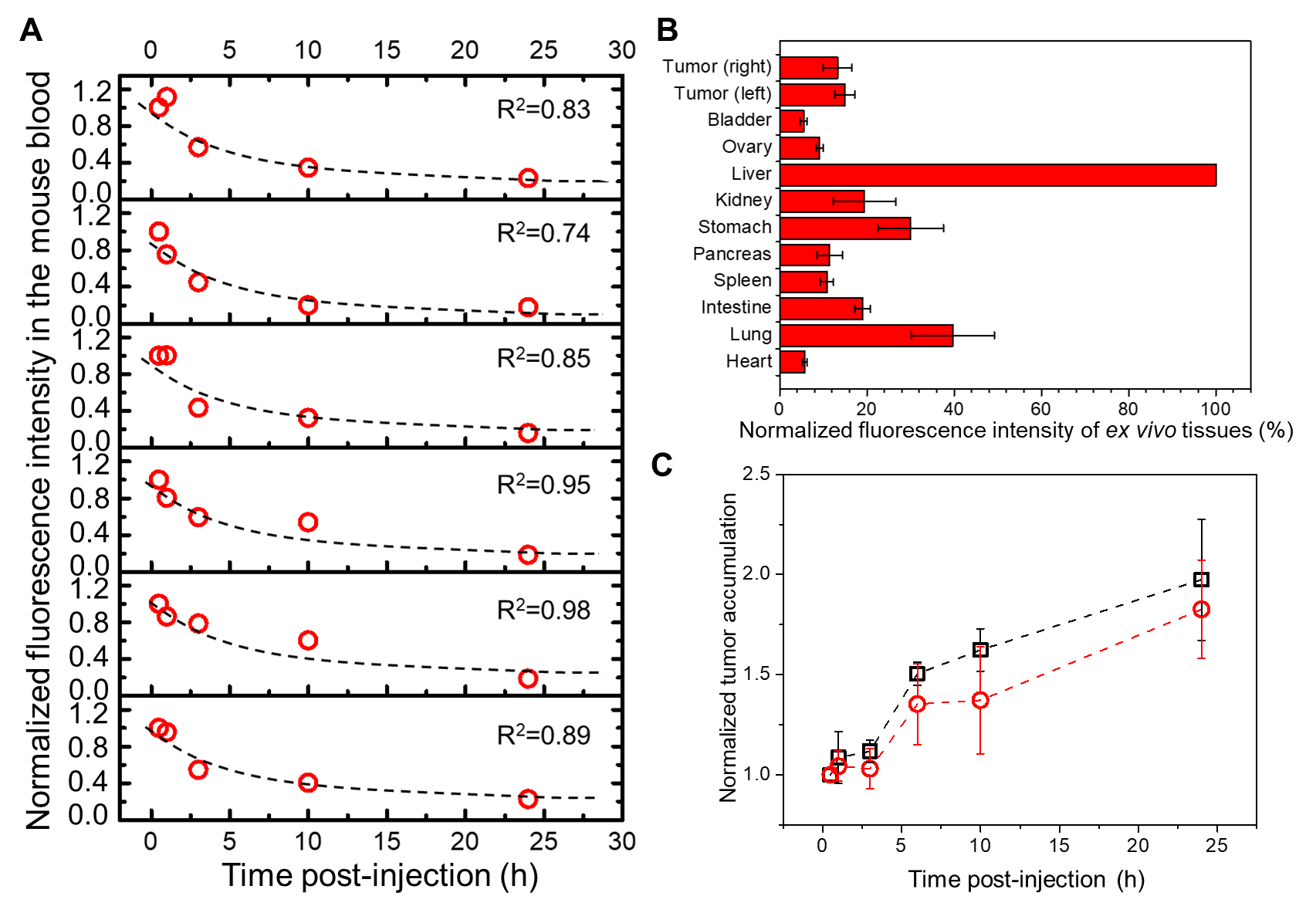
**Figure S5.** *In vivo* profiles of PEO-PCL-P nanoparticles in 8988T cells-transplanted subcutaneous tumor models after tail-vein injection. (A) The fluorescence intensity of mouse blood was plotted versus the withdrawal time, fitting into an exponential function (illustrated as a dotted line in each graph panel), so the exponential decay constant was acquired for conversion into the circulation half-time (t_1/2_). (B) At 24 h p.i., the mice were sacrificed, and the fluorescence intensity of each organ was recorded per tissue area and normalized to that of liver (set to 100%). (C) The fluorescence intensities of both left (black empty squares) and right (red empty squares) tumors were recorded over time, normalized to the first fluorescence reading in each tumor, and plotted against time.


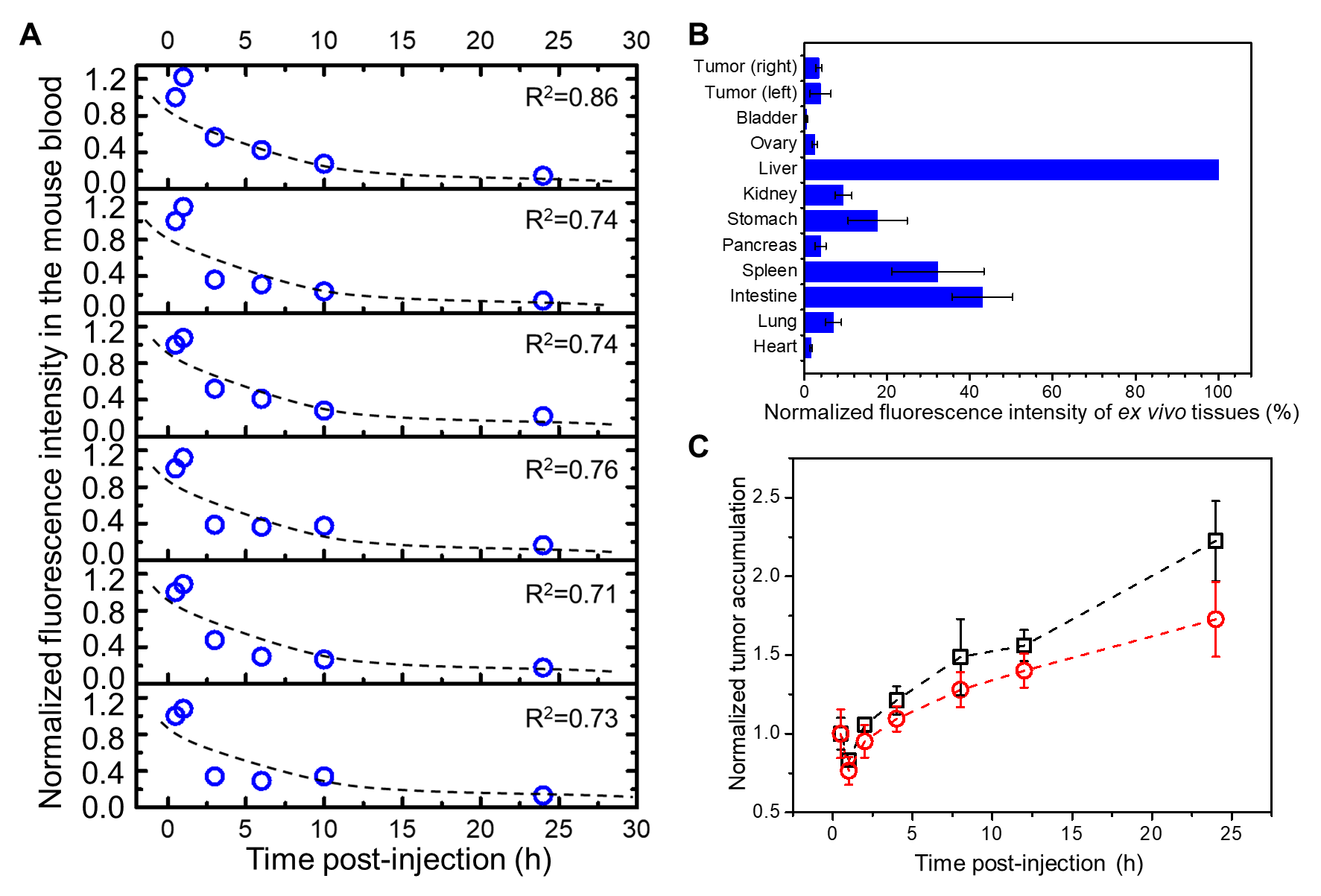
**Figure S6.** *In vivo* results obtained for PEG-DSPE-P nanoparticles in 8988T cells-transplanted subcutaneous tumor models after tail-vein injection. (A) The fluorescence intensity of mouse blood was plotted versus the withdrawal time, fitting into an exponential function (illustrated as a dotted line in each graph panel), so the exponential decay constant was acquired for conversion into the circulation half-time (t_1/2_). (B) At 24 h p.i., the mice were sacrificed, and the fluorescence intensity of each organ was recorded per tissue area and normalized to that of liver (set to 100%). (C) The fluorescence intensities of both left (black empty squares) and right (red empty squares) tumors were recorded over time, normalized to the first fluorescence reading in each tumor, and plotted against time.
